# Birth and Breeding

**Published:** 1882-05

**Authors:** F. M. Holland


### BIRTH AND BREEDING.

F. M. HOLLAND, in *The Index.*

(*Concluded from April Number.*)

V. Fondness for books, especially scientific ones, I found more common among virtuous than vicious people, though the latter show some interest in novels. It is certainly remarkable that of three hundred and twenty criminals in the *Newgate Calendar*, fifty-seven of whom are said to be well educated, but four appear to have cared for reading. The higher the ability, the stronger seems to be this taste, great authors and artists being remarkable for liking poetry and novels, while statesmen, soldiers, and other practical people preferred science.

VI. Early piety and devout parentage seemed much more common among virtuous than vicious people, and the latter were decidedly the more sceptical. Only two devotees appear in the *Newgate Calendar*. Thus there seems to be a strong connection between piety and morality, though it is hard to tell which is the cause and which is the effect. It is simply just, however, to remember that during the last five centuries the power of the Church has steadily declined, but tolerance, philanthropy, chastity, temperance, and respect for laws have greatly advanced. I mentioned that the ratio of crime to population in England is but little more than one third as great now as forty years ago ; but, during that time, church and chapel accommodation has not increased so fast as the number of inhabitants, there has been much legislation hostile to sectarian interests, and secular and rationalistic opinions have spread widely, as will be seen by comparing the earliest and latest volumes of *Punch.*

I feel the more bound to call attention to these facts, because my own figures made the mental influence of the Church appear much less beneficial than the moral. Devout parentage I found to be four times as common among professors of ordinary as of extraordinary mental powers, making all due allowance for moral differences; while early scepticism was twenty-seven times as frequent among people of genius as among those of mediocrity. The ablest men also seem to have been those who most rarely were devotees. No one will be surprised at such indications of the failure of the Church to encourage mental growth, when he remembers that she has preferred faith to free inquiry, emotional to intellectual culture, and aspiration toward a future world to knowledge of the present one, especially in the form of science.

What we think of the Church must however depend mainly, whether we hold that life should be guided by religious enthusiasm or by thoughtful fidelity to the laws of earthly duty.

VII. I must therefore lay stress on the fact that more than twice as large a part of the good people in my lists, as of the bad, exhibited unusual love for business details before the age of thirty, and had great success in making money and gaining promotion; while reckless prodigality and remarkable incapacity of business are characteristic of the vicious. Instances of the murder of creditors by embarrassed debtors abound. One half of the villains in the *Newgate Calendar* had been notoriously idle and extravagant and singularly destitute of ability to make money, so that they had begun to suffer the consequences before they were tempted into crime. Precisely the same is true of the great criminals whose antecedents are minutely recorded by Feuerbach. Juvenile offenders, both in this country and in England, are said to show remarkable incapacity for arithmetic. Dugdale states that: "After disease, the most uniformly noticeable trait of the true criminal is that he lacks the element of continuity of effort. Steady, plodding work is deficient in him." Of six hundred and twenty-five adults in the Bath jail, England, 1848, four fifths were out of work at the time of committing the offence. And so were most of the one hundred and seventy-one discharged soldiers and man-of-war's men who in 1865 were sent to the Massachusetts State's Prison, where only fifteen of them had ever been before. Fletcher, in his laborious study of English statistics, found that where there was the least crime there was not only the most schooling, but the largest number of rich men and the smallest of paupers. Look abroad over the earth's surface, and we find that the most wealthy and industrious countries are those in which liberty is most respected and life and property are most secure. Throughout history, we see commerce, manufactures, just laws, democratic institutions, refinement, temperance, and philanthropy advancing side by side.

These facts lead me to believe that the moral advantages of business habits are much greater than they are usually represented to be, either in popular literature or in the pulpit. For instance, I suspect that for one Ralph Nickelby, Uriah Heep, or James Carker, who becomes a felon, there are ten Dick Swivellers, Bob Sawyers, and Wilkins Micawbers. And I am glad that so few accept literally some well-known texts, which have their value in reminding us that money is not worth so much as virtue, truth, and liberty, but which should not prevent our seeing that these great gifts come to the cool, self-restrained, far-sighted, cautious, yet energetic man of business more readily than to the hot-headed enthusiast.

I am the more bound to insist on the value of business skill, because I find that it has been possessed in the highest degree by about three members out of four in a group of men distinguished as generals, legislators, naturalists, physicians, engineers, inventors, travellers, merchants, or for other forms of practical genius. Of the famous authors and artists not in this group, only one in six had much talent for making money. What is particularly remarkable is that, of the mediocre artists and authors in my lists, twice as large a portion as of the gifted ones showed the highest business ability. Of course, this deficiency is not to be considered as a promoter, but simply as a penalty of genius. Among exceptional cases, the best known are those of Shakespeare and Goethe.

VIII. There was no habit in which I found the best people in various countries differ from the worst so widely and uniformly as in their use of alcohol and tobacco. Excessive indulgence seemed to be eight times as common among the vicious people as among the virtuous; while noticeable, though of course not in all countries complete, abstinence was to the same degree most frequent among the latter. The proportion of drunkards among felons is variously estimated at from thirty-nine to eighty-one per cent, the highest figures being found among juvenile and other petty offenders. The London *Statistical Journal* declares that, with the educated, intoxication is a main cause of crime, increasing in potency as the scale of education rises. Nearly forty per cent of the murders committed in France during a period of four years are ascribed by Quételet to drunken quarrels. The strictest moralists, even in wine countries, have prohibited the use of alcoholic beverages. Among the people whom I found, without special research, to have abstained much more strictly than their friends and neighbors, are the following eminent Europeans : Harriet Martineau, Oberlin, J. J. Gurney, Garibaldi, Robert Owen, Howard, Pestalozzi, J. S. Mill, Comte, Hugh Miller, and D'Alembert. Comparison of mediocre and gifted people showed that early intemperance hinders mental development, especially that of practical genius. Temperate habits may certainly be considered both mentally and morally beneficial.

IX. Fletcher's laborious investigations led him to believe that those parts of England and Wales where the largest proportion of bridegrooms were under twenty-one were the most deeply cursed by ignorance, pauperism, improvidence, licentiousness, and other crime. Dugdale found that his paupers and criminals were remarkable for "precocious sexual excitability," and that the use of ardent spirits was usually preceded and promoted by licentiousness. Nearly one half of the criminals in the *Newgate Calendar* and two thirds of those in Feuerbach's *Trials* had become profligate or jealous before they were thus led into crime. One seventh part of the murders in France are ascribed to these causes. A leading German authority, Oettingen, says that the robber's career usually begins in the house of ill-fame. My own investigations showed that good people are much more apt than bad ones to delay marriage until thirty ; and so are the possessors of literary and artistic genius, especially authoresses. Considering these facts, and also the poverty and ignorance likely to beset the children of improvident marriages, I must say that popular opinion seems to me dangerously to overvalue not only early matrimony, but the goodness of human nature, which appears to me to show traits fully answering to Darwin's theory of its origin. The future of our race demands not only that its healthy, prosperous, virtuous, and intelligent members should marry as soon as they are able to take proper care of their families, but that even they should not do so prematurely, and that the rest should stay as they are.

Passing from these special conclusions to more general ones, the five principal causes of crime appear to be vicious parentage, lack of education from schools and books, inefficiency in business, intemperance, and excessive amorousness. The typical criminal may be described as unsympathetic, obstinate, reckless, fickle, strongly prone to base indulgences, and destitute of taste for books or religious observances. His parentage will be disreputable, his schooling scanty or wholly wanting, his dislike of business great, his behavior ruled by impulses, and all his desires turned toward excitement. His brain will show its strength, if it has any, in fitful flashes, not steady light, and ultimately prove its weakness by sinking into insanity or imbecility.

On the other hand, the moral antecedents of an ideal man seem to be these. His parents will certainly have high character and literary culture, and probably also be both devout and business-like in their habits. His own disposition will be prudent, and at the same time sympathetic. We will gladly learn from others, and yet be able to judge for himself what is right. His lower passions will readily yield to the sway of reason and conscience, and he will be eager to obey the moral laws. His brothers and sisters will have been numerous, his schooling thorough, and his attendance at church in early years constant. He will love books, especially scientific ones, shun stimulants and all other low means of excitement, follow his business steadily and successfully, and not marry prematurely. Hitherto, the highest type of character has almost always been inspired by religious enthusiasm ; but thoughtful and calm adherence to principle seems likely to show that it has even greater power.

And so a great thinker will, I believe, usually be found to have had cultivated parents, æsthetic taste, and thorough education from books, schools, and foreign travel. Though mental ability seems due to peculiarities of brain, which often originate and develop independently of parentage or instruction, literary and artistic genius appears to depend more closely on education, especially through poetry and other forms of art, than practical ability does, this latter seeming to be but little promoted by instruction, except in the form of scientific or business training, and to be mainly due to natural advantages, in part inherited, but to a great extent spontaneous. Prominent among such advantages is a powerful brain which works steadily and regularly and is not excited easily, great susceptibility and excitability being the peculiar accompaniments of literary and artistic genius.

And, finally, nothing seems to me clearer than that greatness, whether mental or moral, depends mainly on innate powers and impulses which are in part inherited, but by no means wholly so. There is only this difference, that the highest goodness may ultimately be reached by whoever will seek it steadily and passionately ; but no strength or persistency of desire for intellectual distinction can possibly achieve it, unless the brain is favorably formed. It depends mainly on natural disposition whether we choose to be saintly ; but, if we do choose, then saints we shall become. We must be born kings of thought, or we shall never wear that crown.

